# Beneficial Impact of CCL2 and CCL12 Neutralization on Experimental Malignant Pleural Effusion

**DOI:** 10.1371/journal.pone.0071207

**Published:** 2013-08-14

**Authors:** Antonia Marazioti, Chrysoula A. Kairi, Magda Spella, Anastasios D. Giannou, Sophia Magkouta, Ioanna Giopanou, Vassilios Papaleonidopoulos, Ioannis Kalomenidis, Linda A. Snyder, Dimitrios Kardamakis, Georgios T. Stathopoulos

**Affiliations:** 1 Laboratory for Molecular Respiratory Carcinogenesis, Department of Physiology, Faculty of Medicine, University of Patras, Rio, Achaia, Greece; 2 First Department of Critical Care and Pulmonary Medicine, University of Athens School of Medicine, General Hospital Evangelismos, Athens, Attica, Greece; 3 Janssen R&D, LLC, Oncology Discovery Research, Spring House, Pennsylvania, United States of America; 4 Department of Radiation Oncology and Stereotactic Radiotherapy, Faculty of Medicine, University of Patras, Rio, Achaia, Greece; Cincinnati Children's Hospital Medical Center, United States of America

## Abstract

Using genetic interventions, we previously determined that C-C motif chemokine ligand 2 (CCL2) promotes malignant pleural effusion (MPE) formation in mice. Here we conducted preclinical studies aimed at assessing the specific therapeutic potential of antibody-mediated CCL2 blockade against MPE. For this, murine MPEs or skin tumors were generated in C57BL/6 mice by intrapleural or subcutaneous delivery of lung (LLC) or colon (MC38) adenocarcinoma cells. Human lung adenocarcinoma cells (A549) were used to induce MPEs in severe combined immunodeficient mice. Intraperitoneal antibodies neutralizing mouse CCL2 and/or CCL12, a murine CCL2 ortholog, were administered at 10 or 50 mg/kg every three days. We found that high doses of CCL2/12 neutralizing antibody treatment (50 mg/kg) were required to limit MPE formation by LLC cells. CCL2 and CCL12 blockade were equally potent inhibitors of MPE development by LLC cells. Combined CCL2 and CCL12 neutralization was also effective against MC38-induced MPE and prolonged the survival of mice in both syngeneic models. Mouse-specific CCL2-blockade limited A549-caused xenogeneic MPE, indicating that host-derived CCL2 also contributes to MPE precipitation in mice. The impact of CCL2/12 antagonism was associated with inhibition of immune and vascular MPE-related phenomena, such as inflammation, new blood vessel assembly and plasma extravasation into the pleural space. We conclude that CCL2 and CCL12 blockade are effective against experimental MPE induced by murine and human adenocarcinoma in mice. These results suggest that CCL2-targeted therapies may hold promise for future use against human MPE.

## Introduction

Malignant pleural effusion (MPE) is a frequent and clinically significant systemic manifestation of various tumors that adversely affects patient survival and quality of life [1], [2]. Etiologic therapies targeting MPE pathobiology are not available, and current treatments, including pleurodesis and indwelling pleural catheters, are evidently symptomatic and suboptimal [3], [4], [5]. However, MPE appears to be precipitated by an array of tumor-to-host signaling events, in addition to lymphatic obstruction of normal pleural fluid outflow [6]. While the biologic pathways that culminate in MPE are progressively unmasked, the possibility of targeted pharmacotherapies against the condition is emerging [7], [8].

We have previously developed animal models of MPE in immunocompetent mice and have identified tumor- and host-originated gene products and host cell populations intimately linked with pleural tumor progression and fluid accumulation [9], [10], [11], [12]. Moreover, we have shown that targeted disruption of biologic pathways that mediate inflammation, angiogenesis, and vascular hyperpermeability during MPE development can yield meaningful improvements in effusion control and survival [13], [14], [15], [16]. Along these lines, we have identified a predominant mononuclear/macrophage cellular infiltrate in experimental and human MPE, and have shown that these cells are recruited to the malignancy-affected pleura by tumor-derived C-C chemokine ligand 2 (CCL2) [11], [17], [18]. In mouse MPE, genetic ablation of CCL2 expression inhibited pleural mononuclear cell accumulation, new vessel formation, and vascular leakage and led to improved outcomes [18]. Although this work identified CCL2 as a promising therapeutic target in preclinical MPE, attempts at suppressing CCL2 signaling using clinically relevant methods have not been undertaken.

In the present study we aimed at therapeutically targeting CCL2 in mouse models of MPE. This was accomplished using proprietary monoclonal antibodies neutralizing mouse CCL2 and/or its murine ortholog, CCL12 [19]. In our hands, treatment of mice with anti-CCL2 and/or anti-CCL12 antibodies alone or in combination inhibited MPE formation in two different syngeneic models. These favorable results were recapitulated in a novel mouse model of human lung adenocarcinoma-caused MPE, indicating that CCL2 blockade may modify the disease course of human MPE.

## Materials and Methods

### Ethics Statement

*C57BL/6J* (hereafter referred to as *C57BL/6*; stock number 000664), severe combined immunodeficient (*NOD.CB17-Prkdc<scid>/J*; hereafter referred to as *SCID*; stock number 001303) and *FVB-Tg(CAG-luc,-GFP)L2G85Chco/J* (hereafter referred to as *CAG.luc.eGFP*; stock number 008450) mice were purchased from the Jackson Laboratory (Bar Harbor, MN). Mice were bred at the Animal Care Facility of the General Hospital Evangelismos (Athens, Greece) and the Center for Animal Models of Disease of the Faculty of Medicine at the University of Patras (Rio, Greece). Three hundred and twenty-one *C57BL/6*, fifty-five *SCID*, and two *CAG.luc.eGFP* mice were used for these studies. Animal care and experiments were approved by the Veterinary Administrations of the Prefectures of Attica and Achaia, Greece (permit numbers: K/4333 and K/7715), and were conducted in strict accordance with EU Directive 86/609/EEC (http://ec.europa.eu/environment/chemicals/lab_animals/legislation_en.htm). All efforts were made to minimize mouse suffering; intrapleural injections were done under isoflurane anesthesia and mice were sacrificed with CO_2_ at the first signs of distress. Moreover, survival experiments were terminated prematurely when end-points of statistical significance were met. Mice used for experiments were sex-, weight (20–25 g)-, and age (6–12 weeks)-matched.

### Cell Lines

Lewis lung carcinoma (LLC) and A549 lung adenocarcinoma cells were purchased from the NCI Tumor Repository (Frederick, MD) and MC38 colon adenocarcinoma cells were provided by Dr. Timothy S. Blackwell (Vanderbilt University, Nashville, TN) [11], [20]. Cell lines were authenticated by the providers using the short tandem repeat method and experiments were done within six months after acquisition. Cells were cultured at 37°C in 5% CO_2_-95% air using Dulbecco’s modified Eagle’s medium (DMEM) supplemented with 10% fetal bovine serum (FBS), glutamine, and 100 mg/l penicillin/streptomycin. For *in vivo* injections, cells were harvested using trypsin, incubated with Trypan blue, and counted as described elsewhere [9], [10], [11]. Only >95% viable cells were used *in vivo*.

### CCL2/12-neutralizing Antibodies

Anti-mouse CCL2 antibody (C1142), anti-mouse CCL12 antibody (C1450A), and negative control mouse antibody (CNTO1322) were provided by Janssen R&D (Spring House, PA). C1142 and C1450A selectively neutralize either mouse CCL2 or mouse CCL12, respectively, and do not neutralize human CCL2 [19].

### Mouse Models of MPE

**Figure 1:**
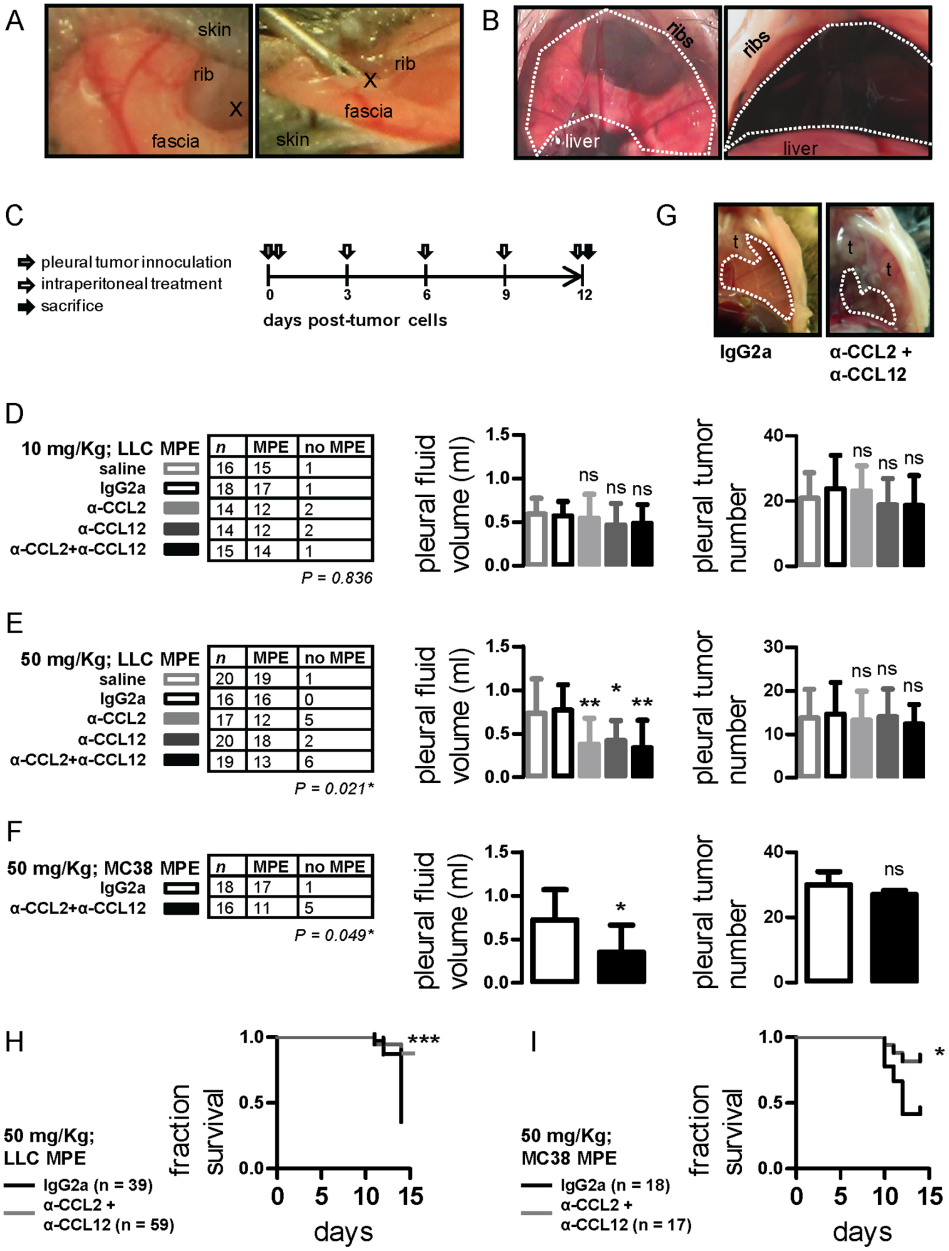
Impact of anti-CCL2 and/or anti-CCL12 monoclonal antibody treatment on syngeneic models of malignant pleural effusion (MPE). (A) Photographs of intrapleural injection technique (x indicates the injection site). (B) Transdiaphragmatic photograph of mouse pleural space before (left) and four hours after (right) intrapleural Evans’ blue delivery. The dashed lines indicate the pleural confines. (C) Graphical outline of *in vivo* experiments. C57BL/6 mice received intrapleural Lewis lung carcinoma (LLC) or MC38 colon adenocarcinoma cells (grey arrow) followed by intraperitoneal treatment with normal saline, IgG2a, anti-CCL2, anti-CCL12, or anti-CCL2 plus anti-CCL12 every three days (white arrows). Mice were terminated after 12 days (black arrow). Primary end-points were MPE incidence and volume. Secondary end-points were pleural tumor number and survival. (D) MPE incidence (left), volume (middle), and pleural tumor number (right) of mice with LLC-induced MPE after regular-dosed antibody treatment. (E) MPE incidence (left), volume (middle), and pleural tumor number (right) of mice with LLC-induced MPE after high-dose antibody treatment. (F) MPE incidence (left), volume (middle), and pleural tumor number (right) of mice with MC38-induced MPE treated with high-dose IgG2a control or anti-CCL2/12 combination regimen. (G) Transdiaphragmatic photographs of representative MC38-induced MPEs from an IgG2a and an anti-CCL2/12 combination-treated mouse. Dashed lines outline MPEs and *t* designates pleural tumors. (H,I) Fractional survival of mice with LLC- and MC38-induced MPE. *Columns*, mean; *bars*, SD; *n*, sample size; *P*, probability by χ^2^ test; ns, *, **, ***: P>0.05, P<0.05, P<0.01, and P<0.001 compared with saline and/or IgG2a control.

For MPE generation, *C57BL/6* mice received 150,000 intrapleural LLC or MC38 cells and SCID mice received 1,000,000 A549 cells in 100 µl PBS. Intrapleural injections were done under direct stereoscopic vision via a small incision in the left anterolateral chest skin and fascia. For this, a 27 G needle was advanced to the pleural space at a 45° angle under direct contact with the superior rib and the tumor cell suspensions were injected under direct visual inspection, as described previously (Figure 1A) [9]. The accuracy of intrapleural tumor cell delivery was tested on three *C57BL/6* mice using Evans’ blue (Sigma, St. Louis, MO): four hours after intrapleural injection of 50 µl Evans’ blue solution (4 mg/ml) the dye was confined to the pleural space (Figure 1B). After pleural tumor cell delivery, mice were randomized to intraperitoneal treatment with normal saline (placebo control), IgG2a (isotype antibody control), anti-CCL2, anti-CCL12, or both anti-CCL2 and anti-CCL12 antibodies in both regular-dose (10 mg/kg) and high-dose (50 mg/kg) protocols. For this, antibodies were delivered in 100 µl normal saline on days 0, 3, 6, 9, and 12 after LLC/MC38 cells and on days 0, 3, 6, 9, 12, 15, 18, 21, 24, and 27 after A549 cells. In MPE control experiments, *C57BL/6* mice were euthanized at day 12 and *SCID* mice at day 28 post-tumor cells. Primary end-point was MPE incidence and volume (MPE control). MPE was diagnosed when pleural fluid volume exceeded 100 µl (the tumor cell injection volume). When no fluid was detected, 20 µl (the pleural fluid volume of naïve mice) was entered into analyses [9]. Exploratory end-points were pleural tumor number, MPE cell number, Evans’ blue leak rate, and survival. For survival experiments, mice were sacrificed when moribund (actual events) or were terminated prematurely (censored events) when intergroup comparison reached the threshold of statistical significance (*P*<0.05) using Kaplan-Meier survival estimates and the log-rank test. Some mice received intravenous injection of 0.8 mg (200 µl; 4 mg/ml) Evans’ blue one hour before sacrifice. MPEs were aspirated via the diaphragm under direct visual inspection till the pleural cavity ran dry by scientists blinded to treatment. MPE volume was measured using gradual pipette aspiration, MPE cells were counted using a hemocytometer, and cell-free MPE supernatants were retrieved after centrifugation at 260 g for 7 min [9], [10]. Pleural tumors were enumerated at autopsy under a Stemi DV4 stereomicroscope (Zeiss, Jena, Germany). Fifty thousand pleural fluid cells were used for cytocentrifugal specimen (cytospin) preparation. Slides were air dried, fixed in methanol for 10 s, and stained with May-Gruenwald-Giemsa. Distinct cell types were enumerated as a percentage of 500 cells on the slide.

### Immunohistochemistry-Immunocytochemistry

Pleural tumors were embedded in optimal cutting temperature medium and frozen, cut into 10-µm-thick cryosections, mounted on glass slides, immunostained using antibodies against proliferating cell nuclear antigen (PCNA; Santa Cruz Biotechnology, Santa Cruz, CA), caspase-3 (Chemicon; Temecula, CA), and factor VIII-associated protein (F8A; Zymed, San Francisco, CA), and counterstained with Hoechst 33258. Immunoreactivity was quantified as described previously [10], [17]. MPE cytospins were stained with anti-CD68 antibody (Abcam, Cambridge, UK) and counterstained with Hoechst 33258, followed by determination of the relative abundance of CD68+ cells.

### CCL2-specific RNA Interference

LLC cells stably transfected with random (non-sense; control) and anti-CCL2-targeted shRNA constructs are described elsewhere [18].

### Tumor Growth Assays

For assessment of *in vivo* tumor growth, LLC or MC38 cells (500,000/50 µl PBS) were injected into the shaved rear flank dermis of *C57BL/6* mice. Thereafter, mice were randomized to intraperitoneal treatment with 50 mg/kg IgG2a or anti-CCL2/CCL12 antibody combination. Antibodies were delivered as above on days 10, 13, 16, 19, 22, 25, and 28 after tumor cells and mice were euthanized at day 28. Three vertical tumor dimensions (δ1, δ2, δ3) were measured weekly and tumor volume (V) was calculated as V = π×(δ1×δ2×δ3)/6 [11]. In similar experiments, *C57BL/6* mice received intradermal LLC cells (500,000/50 µl PBS) expressing random (control) or anti-CCL2-specific shRNAs and tumor growth was monitored as above.

### In vivo Inflammation Monitoring Assay

Ten million luciferase (luc)- and enhanced green fluorescent protein (eGFP)-expressing bone marrow cells were obtained from 4-wk-old *CAG.luc.eGFP* donors and were delivered intravenously to total body-irradiated (1100 Rad) *C57BL/6* mice for endogenous bone marrow replacement [21]. After one month required for full bone marrow and peripheral blood reconstitution [21], baseline bioluminescence imaging was done. Thereafter, animals received intrapleural tumor cells and antibody treatments as above, and were serially imaged for bioluminescence. In this model, bioluminescence stems exclusively from alien bone marrow cells and bioluminescence imaging can be used to monitor immunocyte trafficking.

### Skin Vascular Permeability Assay

*C57BL/6* mice (n = 5) received intradermal injections of IgG2a or cell-free MPE admixed with IgG2a or anti-CCL2 and/or anti-CCL12 antibodies at 50 µg/ml concentration (total injection volume = 50 µl), followed immediately by intravenous delivery of 0.8 mg albumin-binding Evans’ blue. Mice were euthanized after one hour, followed by skin inversion and imaging. The surface area of dye leak was determined using ImageJ software [10], [12], [18], [22]. Each test spot area was normalized to the control test spot on the same mouse.

### Chick Chorioallantoic Membrane (CAM) Angiogenesis Assay

Fertilized chick eggs were fenestrated above the CAM, and the CAM was exposed to IgG2a control or cell-free MPE fluid admixed with IgG2a or anti-CCL2 and/or anti-CCL12 antibodies at 50 µg/ml concentration. Eggs were incubated for 48 hours, followed by CAM removal and imaging, as described elsewhere [23].

### Statistics

All values given represent mean ± SD, since data were normally distributed. Differences between two or multiple experimental groups were compared using χ^2^ test, and Student’s t-test or one-way ANOVA with Tukey’s post-hoc tests, as appropriate. Repeated measures ANOVA was used to evaluate Miles’ assay results. Two-way ANOVA was employed to compare groups of mice with flank tumors. Kaplan-Meier estimates with log-rank tests were employed for survival analyses. All *P* values are two-tailed; *P* values <0.05 were considered significant. All statistical analyses were performed using Prism v5.0.0 (GraphPad, San Diego, CA).

## Results

### CCL2- and CCL12-neutralizing Antibodies are Effective against Experimental MPE Caused by Lung and Colon Adenocarcinoma

To investigate the potential impact of CCL2 and/or CCL12 neutralization on MPE, C57BL/6 mice received intrapleural LLC cells followed by intraperitoneal control or antibody treatments at 10 mg/kg (Figure 1C). Disappointingly, no effect of treatment was observed (Figure 1D). Based on the negative results and the literature, we postulated that higher antibody doses may be required [13], [24]. Indeed, anti-CCL2 and/or anti-CCL12 treatment at 50 mg/kg yielded statistically and biologically significant reductions in effusion incidence and volume compared with saline and IgG2a controls. No differences were observed between the three intervention groups (anti-CCL2, anti-CCL12, and combination treatment). Moreover, the impact of antibody treatment was not associated with reduced intrapleural tumor spread (Figure 1E).

To determine whether the impact of CCL2/12 blockade is applicable to additional tumors, we used another mouse model of MPE formation by MC38 colon adenocarcinoma in *C57BL/6* mice [11]. Indeed, MC38-caused MPE incidence and volume were significantly limited by combined chemokine blockade, despite similar pleural tumor dissemination (Figures 1F, 1G). Improved MPE control was associated with enhancements in survival of CCL2/12-treated mice with both types of syngeneic MPE (Figures 1H, 1I). Collectively, these results indicated that CCL2 and CCL12 neutralization are equally effective against MPE induced by two different mouse adenocarcinomas.

### Anti-CCL2/12 Impact on Tumor Growth

**Figure 2:**
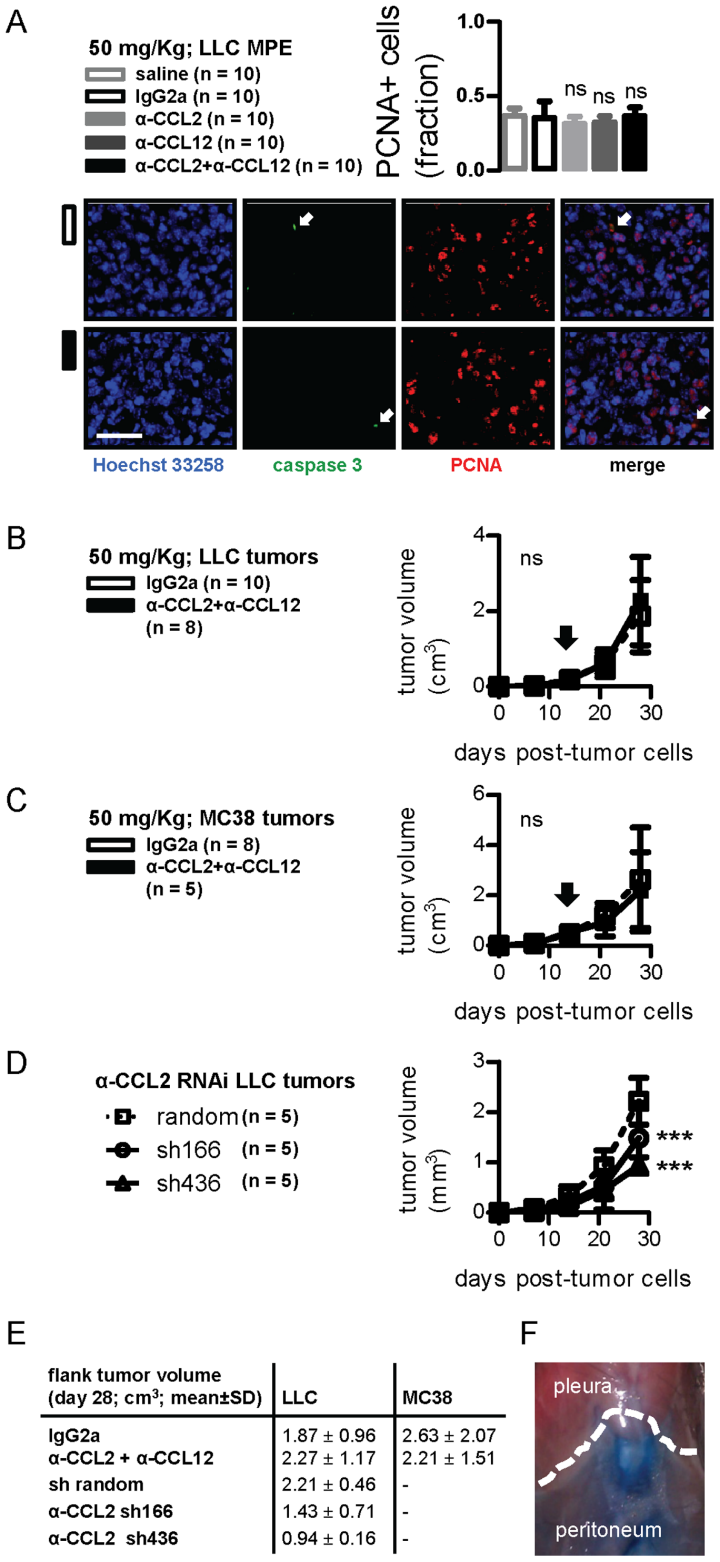
CCL2 and/or CCL12 blockade impact on tumor growth. (A) Pleural tumors from mice with LLC-induced MPE treated as in Figure 1A were stained with Hoechst 33258, anti-Caspase-3, and PCNA antibodies. Shown are summary of data for PCNA staining (left) and representative images from pleural tumors of an IgG2a and a combination-treated mouse (right). Scale bar = 100 µm, Å = 400. Arrows indicate rare caspase-3 positive cells. (B,C) Volume of flank tumors induced by subcutaneous injection of LLC and MC38 cells after treatment with IgG2a control or anti-CCL2/CCL12 combination therapy. Arrows indicate the day of antibody therapy start. (D) Volume of flank tumors induced by subcutaneous injection of LLC cells stably expressing random or anti-CCL2-specific shRNAs (sh166 and sh436). (E) Tumor volumes from flank tumor experiments at four weeks. (F) Photograph of mouse thoracic-abdominal border (dashed line) four hours after intraperitoneal Evans’ blue delivery. *Columns and squares*, mean; *bars*, SD; *n*, sample size; ns and ***, P>0.05 and P<0.001 compared with saline and/or IgG2a or random shRNA controls.

We subsequently tested whether chemokine blockade limits MPE by affecting pleural tumor cell survival. For this, pleural tumor tissue was immune-labeled for PCNA and caspase-3. Almost half of pleural tumor cells displayed nuclear PCNA immunoreactivity, but caspase-3-positive cells were rare. Moreover, no intergroup differences were evident (Figure 2A). In separate experiments, *C57BL/6* mice received subcutaneous LLC or MC38 cells followed by control or antibody combination treatments, but tumor growth rates were identical in both groups (Figures 2B, 2C, 2E). However, CCL2-silenced LLC cells grew significantly slower in the flank dermis compared with control shRNA-expressing LLC cells (Figures 2D, 2E). In search why CCL2/12 blockade was more effective against MPE than flank tumors, we sought to determine whether intraperitoneal injectates communicate directly with the pleural space. Using intraperitoneal Evans’ blue injections, we found no traces of the dye in the pleural space lavage after four hours (Figure 2F and data not shown). These data indicated that CCL2/12 promotes tumor growth in the dermis and, possibly, in the pleural space, but also that CCL2/12 blockade was preferentially effective against MPE.

### Chemokine Blockade Blunts MPE-associated Inflammation

**Figure 3:**
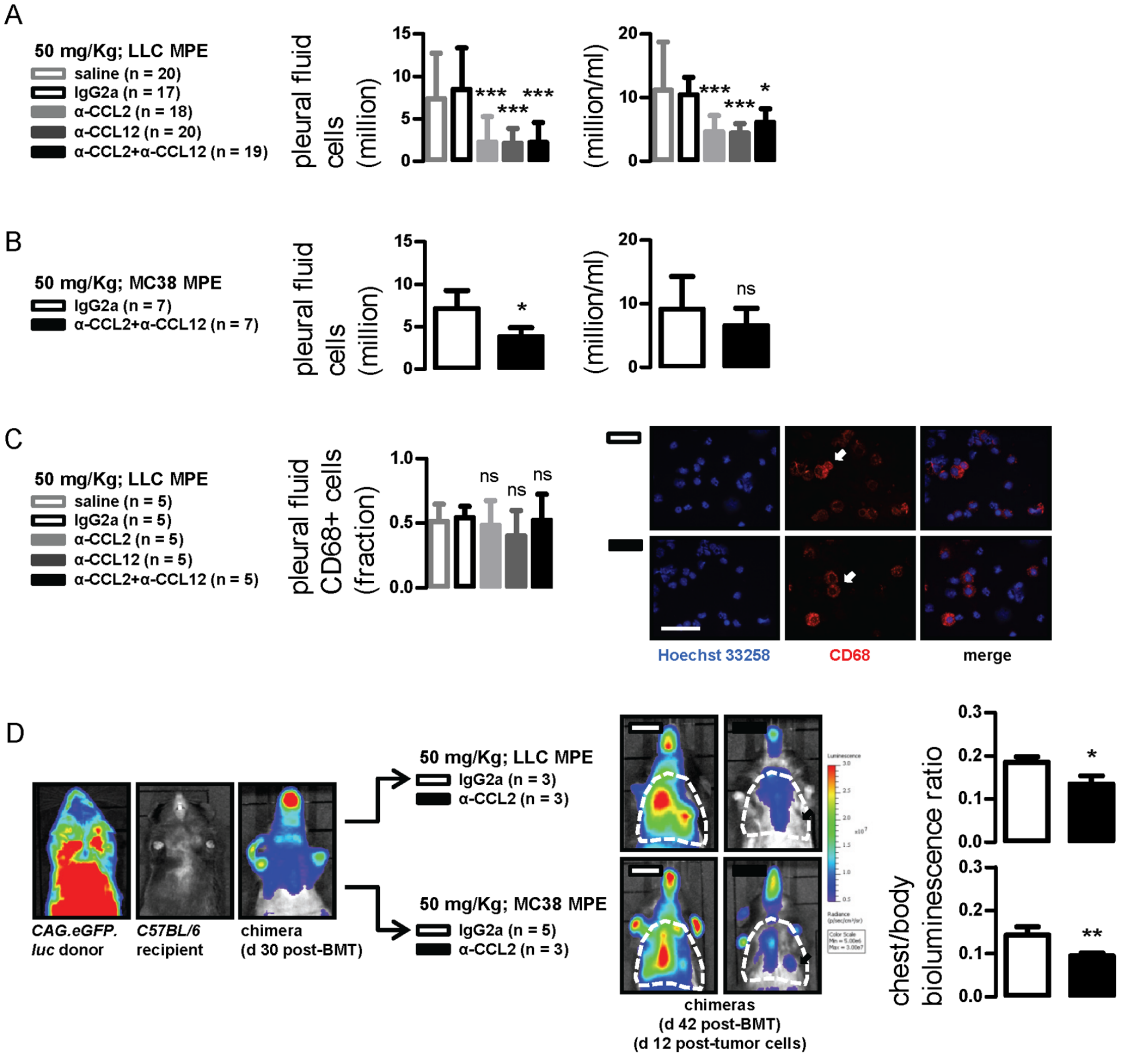
CCL2 and/or CCL12 neutralization limits MPE-associated inflammation. (A, B) Pleural fluid nucleated cells from mice with LLC- and MC38-induced MPE treated as in Figure 1. (C) Results of staining of pleural cells from LLC-induced MPEs for CD68. (D) Representative bioluminescent-photographic image overlays (left) and summary of data obtained from C57BL/6 mice adoptively transplanted with labeled bone marrow obtained from CAG.luc.eGFP-donors. In this model that can be used to monitor inflammation, bioluminescence stems exclusively from alien bone marrow transplants. Chimeric mice were imaged for light emission before and after MPE induction followed by IgG2a or anti-CCL2/12 combination treatment. Increased thoracic (dashed outlines) bioluminescence was observed after MPE induction, reflecting the inflammatory response that develops with MPE. The phenomenon was markedly inhibited in mice treated with anti-CCL2/12 antibody combination (arrows). *Columns*, mean; *bars*, SD; *n*, sample size; ns, *, **, and ***: P>0.05, P<0.05, P<0.01, and P<0.001 compared with saline and/or IgG2a control.

In search for additional effects of our treatment, we sought to assess MPE-related inflammation in our experimental mice. Pleural fluid nucleated cell number and type were not altered by antibody treatment at 10 mg/kg in the LLC model (data not shown). In contrast, a robust reduction in the absolute and relative abundance of MPE cells was observed using 50 mg/kg (Figure 3A). Interestingly, no intergroup differences in the relative abundance of mononuclear, polymorphonuclear, or lymphoid cells were evident by morphology (data not shown). A decrease in absolute but not relative MPE cellularity was also found in mice with MC38-caused MPE treated with anti-CCL2/12 antibody combination compared with IgG2a-treated controls (Figure 3B), but no differences in inflammatory cell types were evident (data not shown). Since CCL2 is important for mononuclear cell recruitment, we immunolabeled pleural fluid cells from LLC-induced MPEs for CD68, a comprehensive pleuropulmonary mononuclear marker [25]. Again, no differences in the relative abundance of CD68+ cells were found (Figure 3C). Collectively, the data indicated that CCL2/12 neutralization inhibited overall inflammatory cell recruitment to the pleural space without predilection for a given cell type.

To verify this using an integral approach, we developed a model to monitor MPE-triggered inflammation in the thoracic cavity. Specifically, we ablated the native bone marrow of *C57BL/6* mice by total body irradiation and reconstituted them with same-day bone marrow transfer from *CAG.luc.eGFP* donors, as described previously [21]. One month later, a time-point where full reconstitution of chimeric mice with alien bone marrow had occurred (data not shown and reference [21]), mice were imaged for bioluminescence. Photon emission originated from the whole body, but was strongest over bony structures and weak over the thorax (Figure 3D; chimera d 30 post-BMT). Mice then received intrapleural LLC or MC38 cells followed by 50 mg/kg IgG2a or anti-CCL2/12 combination and serial bioluminescent imaging. At day 12 post-tumor cells (day 42 post-BMT), a clear-cut bioluminescent signal increase was evident over the chest of IgG2a-treated controls, but was significantly suppressed in anti-CCL2/12-treated mice (Figure 3D; images on the right and graphs). Collectively, these data indicated that CCL2 and CCL12 blockade significantly blunts MPE-associated inflammation, a cardinal constituent of MPE pathogenesis. Moreover our findings identified an experimental model to monitor tumor-related inflammation in the living mouse.

### CCL2/12 Neutralization Inhibits Vascular Events Critical for MPE Development

**Figure 4:**
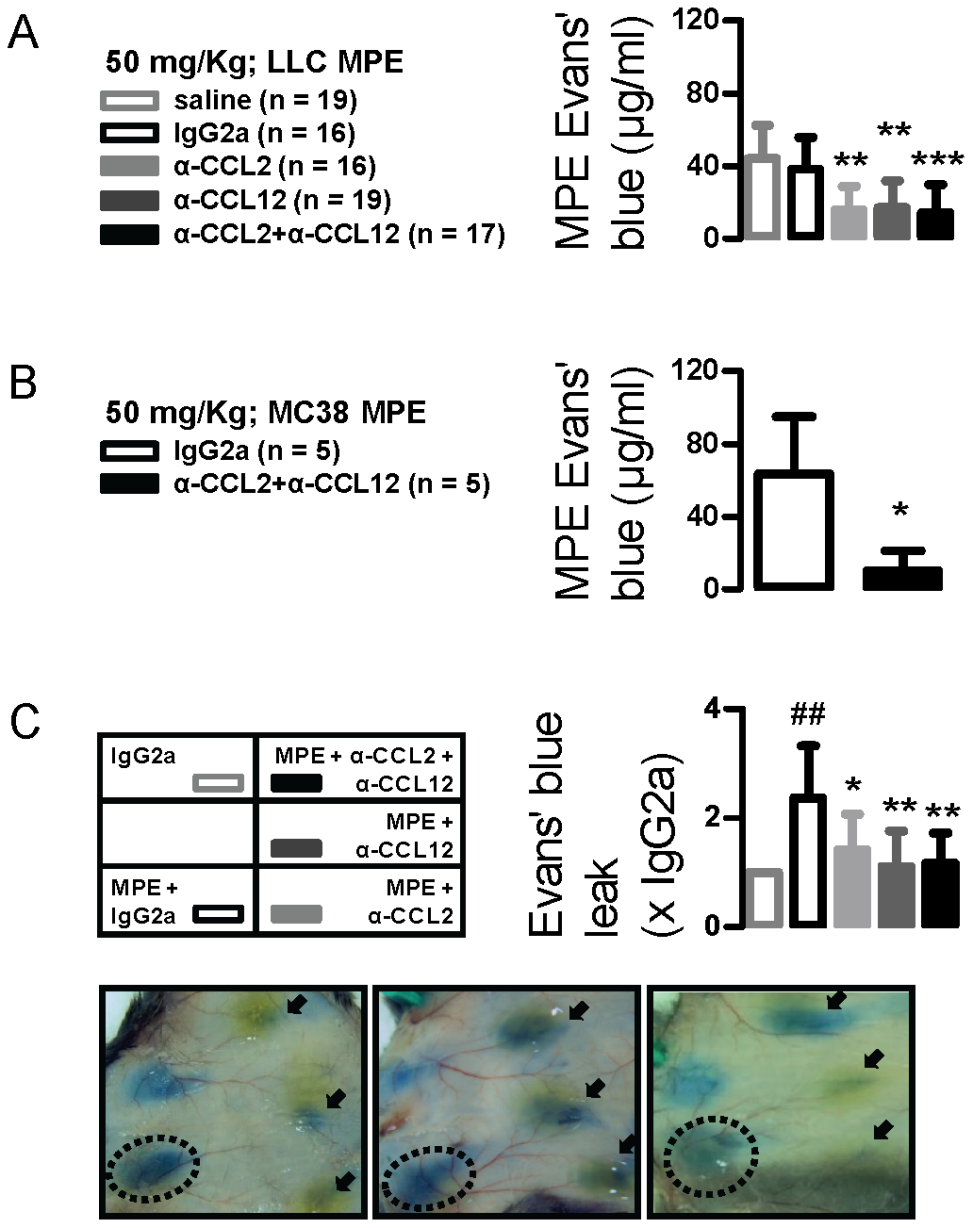
CCL2 and/or CCL12 neutralization inhibits MPE-precipitating vascular hyperpermeability. (A,B) Pleural fluid Evans’ blue levels from mice with LLC- and MC38-induced MPE treated as in Figure 1A. Mice received intravenous Evans’ blue before sacrifice, followed by quantification of the albumin tracer in MPE. *, ** and ***: P<0.05, P<0.01, and P<0.001 compared with saline and/or IgG2a. (C) Summary of data (*n* = 5) and photographs of representative skin test sites from C57BL/6 mice that received intradermal injections of IgG2a or cell-free MPE fluid admixed with IgG2a or anti-CCL2/12 antibodies, followed immediately by intravenous delivery of Evans’ blue. Mice were euthanized after one hour, followed by skin inversion and imaging. Shown is test spot area relative to the control test spot on the same mouse. ##: P<0.01 compared with IgG2a; * and **: P<0.05 and P<0.01 compared with MPE. *Columns*, mean; *bars*, SD; *n*, sample size.

Leakiness of juxtapleural blood vessels is key to clinical and preclinical MPE formation [6]. To study it, we pulsed MPE-bearing mice treated with control or anti-CCL2/12 antibodies with intravenous albumin-binding Evans’ blue and measured it in MPE fluid after one hour [9], [10]. MPE Evans’ blue levels were not affected by antibody treatment at 10 mg/kg (data not shown). However, the levels of the albumin tracer were ∼60% reduced in LLC-MPE-bearing mice treated with either or both intervention antibodies at 50 mg/kg compared with controls (Figure 4A). An even greater effect (>80% reduction) was observed in the MC38 model (Figure 4B). These results were corroborated using a modified Mile’s assay, where cell-free MPE induces vascular hyperpermeability in the mouse dermis [9], [18]. Use of anti-CCL2/12 antibodies in this system resulted in marked inhibition of Evans’ blue extravasation (Figure 4C). These data indicated that CCL2 and CCL12 blockade limits MPE-associated vascular hyperpermeability, the event directly preceding MPE formation.

**Figure 5:**
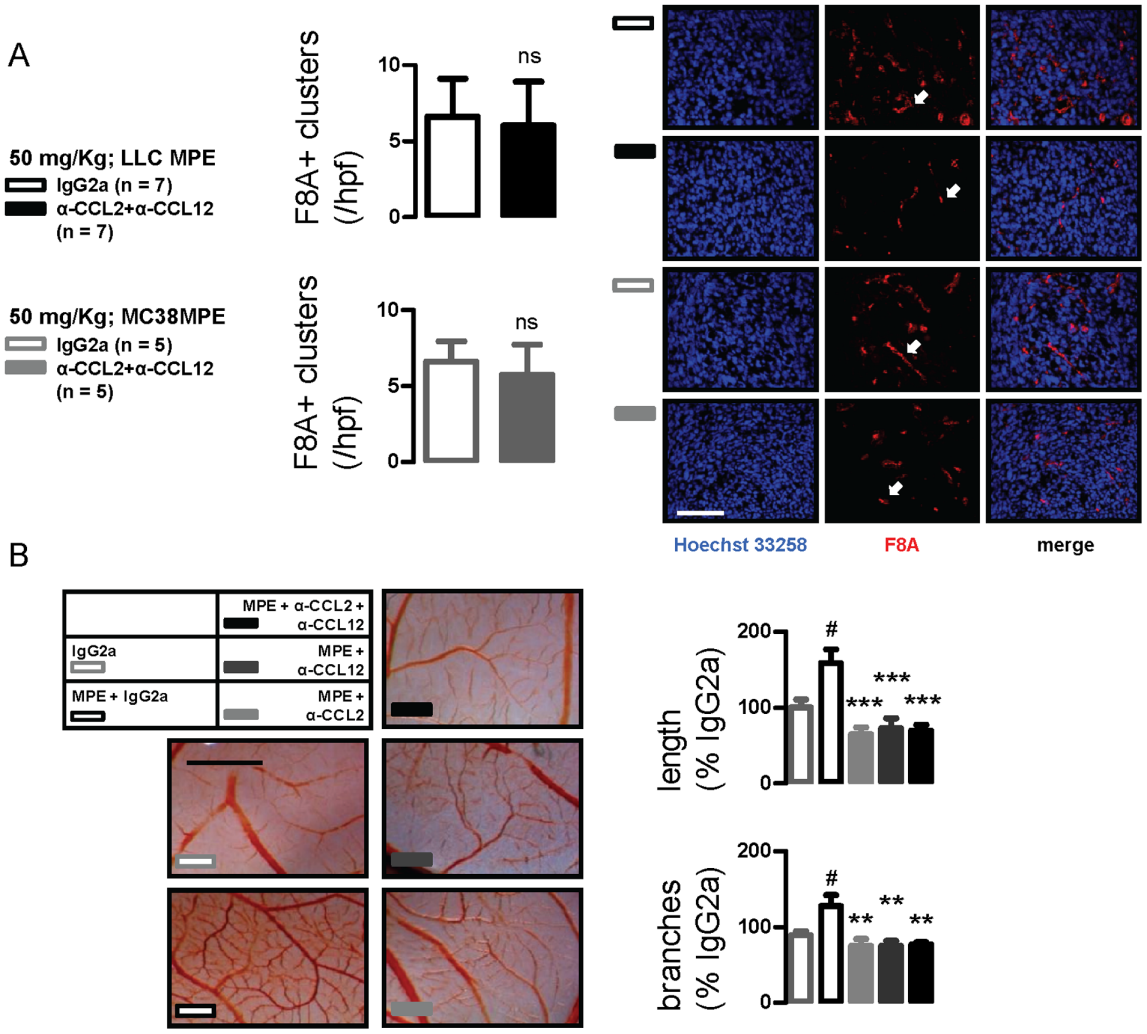
CCL2 and/or CCL12 neutralization impacts new vessel assembly in pleural tumors. (A) Microvessel density of pleural tumors from mice with LLC- and MC38-induced MPE treated with IgG2a or anti-CCL2/12 combination as in Figure 1A. Shown is summary of data and representative images of factor-VIII-associated protein (F8A) immunoreactivity. Scale bar = 100 µm; Å = 400. Arrows indicate new vessels. (B) Representative chorioallantoic membranes and summary of data obtained from six membranes/group that were incubated with IgG2a or cell-free MPE fluid admixed with IgG2a or anti-CCL2/12 antibodies. Scale bar = 5 mm. *Columns*, mean; *bars*, SD; *n*, sample size; ns: P>0.05; #: P<0.05 compared with IgG2a; *, **, and ***, P<0.05, P<0.01, and P<0.001 compared with MPE.

We next examined pleural tumors for newly formed blood vessels by immunolabeling for F8A [10], [17], [18]. Pleural tumor microvessel density was not different between IgG2a- and anti-CCL2/12 combination-treated mice with LLC- or MC38-caused MPEs; however, blood vessels in CCL2/12-targeted mice appeared smaller and less organized (Figure 5A). In the CAM model of angiogenesis [23], cell-free MPE was able to stimulate neovessel formation within 48 hours, and this increase was abrogated by anti-CCL2 and anti-CCL12 antibodies. Similar to pleural tumors, newly formed microvessels in chemokine-targeted CAMs appeared hazy and less well-organized (Figure 5B). These findings suggested that CCL2/12 signal inhibition retards neovascular assembly during MPE development.

### Anti-CCL2 Treatment is Effective against Human Lung Adenocarcinoma-triggered MPE: Contributions of Host-derived CCL2

**Figure 6:**
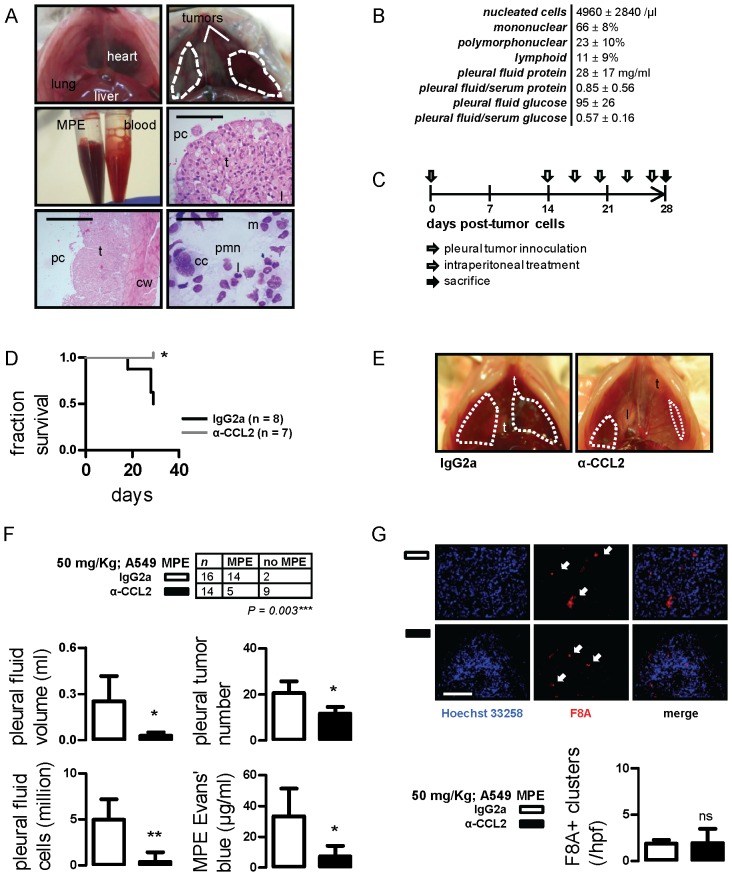
CCL2 blockade limits MPE induced by A549 human lung adenocarcinoma. (A) Development of a novel model of MPE induced by A549 cells. Shown are transdiaphragmatic photographs of SCID mouse indicating the normal anatomy (top left) and SCID mouse with A549-induced MPE (top right; dashed lines); retrieved MPE and blood samples (middle left); hematoxylin & eosin-stained visceral (middle right; scale bars = 400 µm; Å = 100) and parietal (bottom left; scale bars = 800 µm; Å = 50) pleural tumor tissue sections indicating the pleural cavity (pc), tumors (t), lung (l) and the chest wall (cw); and May-Gruenwald-Giemsa-stained pleural fluid cell cytocentrifugal specimen (scale bar = 100 µm; Å = 400) showing a mixture of cancer (cc), mononuclear (m), polymorphonuclear (pmn), and lymphoid (l) cells (bottom right). (B) Cellular and biochemical composition of A549-induced MPE in SCID mice (values given represent mean ± SD; n = 10). (C) Experimental set-up of host-directed anti-CCL2 trial using the xenogeneic A549/SCID MPE model. (D) Kaplan-Meier survival curve of SCID mice after intrapleural delivery of A549 cells followed by IgG2a or anti-CCL2 antibody treatment. (E,F) Representative transdiaphragmatic photographs and experimental end-points of A549/SCID MPE trial. Dashed lines outline MPEs; *t*, tumor; *l*, lung. (G) Summary of data and representative images of F8A-stained pleural tumors from the A549/SCID MPE model. Scale bar = 100 µm; Å = 400. Arrows indicate new vessels. *Columns*, mean; *bars*, SD; *n*, sample size; *P*, probability; ns, *, and **: P>0.05, P<0.05, and P<0.01 compared with IgG2a.

We postulated that, in addition to tumor-originated CCL2, chemokine elaborated by host cells contributes to MPE development. To study this and to translate our findings to a setting more relevant to human MPE, we injected A549 cells into the pleural space of SCID mice (*n* = 10). After 28 days, all mice developed bloody pleural effusions that did not coagulate *ex vivo* and pleural adenocarcinomas that invaded the visceral and parietal pleura. In the pleural fluid, a mixed inflammatory infiltrate coexisted with cancer cells, confirming the malignant nature of these effusions (Figure 6A). Inflammatory cells were predominantly mononuclear, followed by polymorphonuclear and lymphoid cells. Compared with matched serum, A549 MPEs had high protein content and would be classified as exudates according to Light’s criteria, and low glucose levels similar to many human MPEs (Figure 6B) [26], [27]. In addition, A549 effusions were lethal: median survival was 33 (95% confidence interval: 30–37) days.

To test the role and druggability of host-derived CCL2 in MPE caused by human cancer, SCID mice received intrapleural A549 cells followed by IgG2a or anti-CCL2 antibody treatments (Figure 6C). In this model, tumor-elaborated CCL2 cannot be neutralized by our murine-specific anti-CCL2 antibodies [19]. CCL2 blockade yielded meaningful benefits, including significantly prolonged survival (Figure 6D), as well as marked reductions in MPE incidence and volume, pleural tumor dissemination, and markers of pleural inflammation and vascular leakage (Figures 6E, 6F). Immunodetection of murine F8A in A549 pleural tumors turned out similar with syngeneic studies: no intergroup difference in microvessel density was established, but neovessels appeared smaller and less organized in CCL2-targeted mice (Figure 6G). These results showed that, in addition to murine MPE, CCL2 neutralization conferred benefit against human tumor-induced MPE. Moreover, the data indicated that host-originated CCL2 contributes to MPE development.

## Discussion

In the present study we investigated the therapeutic potential of CCL2- and/or CCL12-neutralizing antibodies against experimental MPE. Building on a previously reported cardinal role for CCL2 in MPE pathobiology [18], we show here that CCL2/12 blockade using a clinically relevant intervention limits pleural fluid accumulation in two syngeneic mouse models. Our data indicate that high antibody doses are required to achieve preclinical benefit. Importantly, although chemokine neutralization was not associated with hampered tumor growth, it suppressed tumor effects on the host vasculature and immune system. To validate our results in a setting closer to human MPE, we developed a xenogeneic model of MPE induced by human lung adenocarcinoma. Mouse-specific CCL2 neutralization showed significant efficacy in this model, recapitulating the data from syngeneic models and showing significant contributions of host-derived CCL2 in MPE biology. Collectively, our results indicate that CCL2 and CCL12 neutralization alone or in combination favorably impact MPE development in mice and support that MPE control can be achieved by targeting tumor-host interactions rather than tumor cell survival.

Human CCL2 has two murine orthologues: CCL2 and CCL12 both signal via CCR2 to affect inflammatory cell trafficking, mainly of the mononuclear lineage [28]. We found that neutralizing either or both chemokines yielded identical effects on LLC-induced MPE formation. This observation, in agreement with previous reports [29], led us to adopt combined neutralization in subsequent experiments. In the A549 model, we used the anti-CCL2 antibody only, to gain mechanistic insights into host-CCL2 effects. In this regard, the anti-CCL2 antibody we used has been shown to neutralize only mouse CCL2 [19]. In addition, we and others have previously shown that host cells, such as macrophages, can be a significant source of CCL2 [17]. Taken together, our data show that targeting of both tumor and host-secreted CCL2 (or its orthologue, CCL12) in different MPE models yielded similar results, namely inhibition of MPE induction.

The existing evidence indicates that development of MPE, a clinically important and mechanistically intriguing phenotype of various cancers, does not depend merely on cell-autonomous tumor hallmarks [6]. Current doctrines suggest two major mechanisms of MPE development: tumor-induced obstruction of pleural fluid removal synergizes with tumor-induced plasma extravasation into the pleural cavity resulting in net fluid accumulation [6], [30]. For the latter to be accomplished, tumor cells signal to the juxtapleural vasculature and immune system to induce endothelial proliferation, leakage, and further recruitment of inflammatory cells [6]. We have previously shown that CCL2 is an MPE-effector capable of recruiting mononuclear cells to the pleural space, of inducing angiogenesis, and of rendering blood vessels leaky [18]. The present work corroborates CCL2 as a culprit for MPE and establishes the chemokine as an attractive therapeutic target. Equally importantly, the data strengthen the above mechanistic views on MPE pathobiology: in our hands anti-CCL2/12 effects were not restricted to inhibiting tumor growth, but included blunting of emergent tumor-to-host signaling events resulting in reduced recruitment of inflammatory cells to the malignancy-affected pleura, disruption of pleural tumor vasculature, and down-regulated permeability of juxtapleural blood vessels.

Tumor-associated inflammation is an important hallmark of cancer that enables the acquisition or manifestation of specific tumorigenic phenotypes [31]. Here we demonstrate that blockade of CCL2/12 in immune-intact mouse models of MPE alters disease progression by inhibiting inflammatory cell accumulation in the pleural microenvironment. Moreover, we describe an experimental setup that allows for real-time monitoring of tumor-related inflammation using bioluminescence imaging. In mice with MPE, we did not identify a distinct inflammatory cell population that is dependent on chemokine signaling, and all MPE cell types were decreased in CCL2/12-targeted mice. In this regard, tumor-associated inflammatory infiltrates are almost invariably mixed indicating coordinated, sequential recruitment of different inflammatory cells to the tumor milieu [32]. In addition, CCL2 signaling impacts mononuclear, myeloid suppressor, and lymphoid cell biology and current evidence indicates functional and morphologic diversity even of immune cells previously considered distinct [33], [34], [35]. For example, myeloid suppressor cells, known to be recruited to tumor sites by CCL2, were recently shown to appear as both monocytes and neutrophils [36]. Taken together, our data indicate that CCL2/12 blockade globally limits MPE-enabling inflammation in the pleural space, disabling the pleural-directed trafficking of multiple immune cell types.

In the malignancy-affected pleura, inflammation potentiates abnormally increased vascular permeability to plasma proteins and to circulating blood cells [6], [37]. We and others have previously shown that this enhanced pleural-directed extravasation is the hallmark of MPE, is required and sufficient for MPE formation, and is provoked by mediators such as vascular endothelial growth factor, tumor necrosis factor, and CCL2, among others [6], [10], [18], [38]. More importantly, this pathogenic hyperpermeability may *per se* augment MPE-associated inflammation and is treatable: interventions tailored to block inflammatory signaling can abrogate aberrant vascular leakiness and can limit MPE formation [6], [9], [10], [12], [13], [14], [15], [39]. Here, using Evans’ blue pulse-and-chase, we show that CCL2/12 blockade is such a strategy to hamper enhanced extravasation in mice with pleural tumors. In addition, CCL2 and/or CCL12 neutralization prevented MPE-induced permeability in the murine dermis. These results provide a potential direct mechanism of action of CCL2/12 blockade against MPE. Since CCL2 neutralization suppressed human adenocarcinoma-associated pleural vascular permeability, this approach could also be effective against human MPE.

During MPE development, aberrant vascular leakiness is not restricted to normal subpleural capillaries, but also occurs in new vessels that are formed within pleural tumors [6], [40]. Based on the above, we sought to determine whether the reductions in vascular permeability achieved by anti-CCL2/12 antibodies were due to functional changes of the endothelial barrier, or could also be attributed to structural changes induced by our treatment to the pleural tumor neovasculature. In mouse and human cancer-originated pleural tumors, we observed that microvascular density was not affected by our treatment. However, a qualitative impact of CCL2/12 blockade was evident: blood vessels within pleural tumors appeared smaller and less well-organized in chemokine-targeted animals, indicating retardation of neovascular assembly. To clarify this, we utilized a simpler *in vivo* model, where angiogenesis occurs rapidly and two-dimensionally: in the CAM, CCL2 and/or CCL12 neutralization equally inhibited vessel branching, elongation, and organization within 48 hours, verifying its anti-angiogenic effects and explaining the results from the animal models. CCL2 has been shown previously to function as a direct and indirect mediator of angiogenesis. Signaling via the CCR2 receptor in endothelial cells, it promotes the proliferation, migration, and assembly of endothelial cells into capillary-like structures [41]. Collectively, our results indicate that, in addition to inflammation and vascular leakage, targeting of proinflammatory CCL2/12 limits new vessel formation in the metastatic pleural microenvironment. Together with recent data that clearly show the contribution of inflammatory myeloid cells to the endothelial cell population of tumors and the close interconnectedness of inflammation and angiogenesis, clinical targeting of CCL2-dependent inflammation may prove to be an effective anti-angiogenic therapy [34], [42].

In our hands, CCL2 and/or CCL12 neutralization did not alter pleural tumor dissemination and subcutaneous tumor growth. These results are different from data reported from other models and tumors, where CCL2 blockade impacts tumor growth [29], [36]. However, genetic ablation of CCL2 expression in LLC cells resulted in reduced tumor growth. There are several possible reasons for this discrepancy. First, antibody kinetics, dosing and timing may have different characteristics in mice with MPE and solid tumors. Second, CCL2 may promote metastatic tumor growth only in tumors and models where the chemokine facilitates metastatic homing, i.e., a chance for tumor cells to grow [42], [43]. Third, CCL2 may selectively promote tumor growth in models that require CCL2-mediated immune evasion [36], in contrast to MPE where CCL2 apparently functions as an escalator of frank inflammation. Finally, pleural fluid accumulation and not solid pleural tumor growth likely presents the dominant manifestation of tumor progression in the mouse models of MPE, as opposed to solid tumors where tumor cell proliferation outweighs interstitial fluid accumulation [44]. Taking into account the above observations, one may be less frustrated by the failure of anti-CCL2 antibody against human metastatic castration-resistant prostate cancer, a phase II study where treatment was actually well-tolerated [45]: CCL2-dependent tumors and cancer phenotypes may be defined and are yet to be discovered. Collectively, published results and the data from our studies suggest that CCL2 may or may not exert tumor growth-promoting effects depending on the host environment. More importantly, the available evidence indicates that targeting of the chemokine disrupts cardinal tumor-host signaling events and yields conditional preclinical benefit independent from direct tumor toxicity.

### Conclusions

Here we show that two available CCL2 and CCL12 antagonists alone or in combination are effective in limiting MPE induced by murine and human adenocarcinomas. The data support that CCL2/12 blockade functions to block emergent cancer hallmarks of specific importance to pleural fluid production, such as inflammation and vascular homeostasis. From a clinical perspective, our findings highlight the need for further validation of anti-CCL2-directed therapies against malignant pleural disease.
